# Anisotropy of Additively Manufactured Metallic Materials

**DOI:** 10.3390/ma17153653

**Published:** 2024-07-24

**Authors:** Binghan Huangfu, Yujing Liu, Xiaochun Liu, Xiang Wu, Haowei Bai

**Affiliations:** Institute of Metals, College of Materials Science and Engineering, Changsha University of Science & Technology, Changsha 410004, China; binghanhuangfu@163.com (B.H.); xcliu@csust.edu.cn (X.L.); csustwuxiang@163.com (X.W.); bernkastel01g@gmail.com (H.B.)

**Keywords:** additive manufacturing, thermal-affected zone, anisotropy

## Abstract

Additive manufacturing (AM) is a technology that builds parts layer by layer. Over the past decade, metal additive manufacturing (AM) technology has developed rapidly to form a complete industry chain. AM metal parts are employed in a multitude of industries, including biomedical, aerospace, automotive, marine, and offshore. The design of components can be improved to a greater extent than is possible with existing manufacturing processes, which can result in a significant enhancement of performance. Studies on the anisotropy of additively manufactured metallic materials have been reported, and they describe the advantages and disadvantages of preparing different metallic materials using additive manufacturing processes; however, there are few in-depth and comprehensive studies that summarize the microstructural and mechanical properties of different types of additively manufactured metallic materials in the same article. This paper begins by outlining the intricate relationship between the additive manufacturing process, microstructure, and metal properties. It then explains the fundamental principles of powder bed fusion (PBF) and directed energy deposition (DED). It goes on to describe the molten pool and heat-affected zone in the additive manufacturing process and analyzes their effects on the microstructure of the formed parts. Subsequently, the mechanical properties and typical microstructures of additively manufactured titanium alloys, stainless steel, magnesium–aluminum alloys, and high-temperature alloys, along with their anisotropy, are summarized and presented. The summary indicates that the factors leading to the anisotropy of the mechanical properties of metallic AM parts are either their unique microstructural features or manufacturing defects. This anisotropy can be improved by post-heat treatment. Finally, the most recent research on the subject of metal AM anisotropy is presented.

## 1. Introduction

Metal additive manufacturing (AM), commonly known as metal 3D printing, is a rapid prototyping technology. Originating in the 1980s, AM is an advanced manufacturing technology that integrates mechanics, computer science, numerical control, and materials [1,2]. The basic principle of 3D printing technology is to manufacture parts or prototypes by accumulating layers based on two-dimensional cross-sectional information obtained from slicing three-dimensional solid parts. This process uses points, lines, or surfaces as fundamental units to achieve the final solid part or prototype [3,4,5,6]. AM differs from traditional subtractive methods (such as machining) and formative methods (such as forging) and enables the production of complex structures that are difficult or impossible to achieve with conventional methods. Additionally, AM significantly reduces the number of processing steps and shortens the manufacturing cycle [7,8,9,10]. Its notable advantages have led to its recognition as the core technology of the “Third Industrial Revolution [11]”. The continuous advancement of information technology innovation is leading to a new stage of intelligence and digitalization in industrial production. It is likely that, in the future, this will result in better control of processing variables in the manufacturing process. Germany already proposed the ‘Industry 4.0’ development plan in 2014, which is likely to result in significant disruption and innovation within the industrial sector. It is anticipated that additive manufacturing technology will become a prominent driver of industrial intelligence development. Many scientists have indicated that metal AM has the potential to completely transform the design and manufacturing processes of metal components in the digital industrial era. Therefore, in the past decade, the use of AM technology to produce metallic parts has increased significantly. Correspondingly, the industrial application of metal AM production technology has also become more mature. For these reasons, many scientists have extensively researched metal-formed parts produced using AM technology. Despite the many advantages of AM technology over traditional processes, it is essential to note that additive manufacturing will not replace traditional manufacturing for a long time. Rather than being competitors, the two manufacturing methods are more complementary. If enterprises use AM technology to design parts for production and create products, the related AM industrial chain companies can generate substantial value and good returns.

Additive manufacturing (AM) is a novel manufacturing process that offers a multitude of advantages over traditional removal-based machining [12,13,14,15,16]. AM builds complex three-dimensional structures directly from digital models by stacking materials layer by layer, thus providing a wide range of applications. Firstly, the design freedom and complexity that additive manufacturing technology offers represents a significant departure from the constraints imposed by traditional manufacturing techniques [17,18]. This allows designers to achieve a level of freedom and complexity that was previously unattainable. The use of computer-aided design (CAD) software enables designers to create complex geometries and structures with precision, which in turn facilitates the generation of more optimized functional designs. This flexibility not only enhances the performance of the product, but also reduces the necessity for assembly and connections in traditional manufacturing, thereby streamlining the overall manufacturing process. Secondly, metal additive manufacturing technology can reduce the time and resources required for production [19,20,21,22]. In contrast to the wasteful cutting processes common in traditional manufacturing, additive manufacturing technology minimizes material waste by precisely controlling the addition of material. This not only reduces the cost of raw materials, but also contributes to environmental sustainability. Furthermore, additive manufacturing has the capacity to utilize scrap or recycled materials [23,24], thereby reducing production costs and the environmental impact. Concurrently, additive manufacturing technology facilitates expeditious product development and manufacturing in a relatively brief period, considerably enhancing production flexibility and responsiveness. This is of particular importance in the context of rapidly evolving market demands and the growing trend toward personalization. Manufacturers can rapidly produce customized products according to customer needs [25], thereby enhancing customer satisfaction and market competitiveness. Finally, despite the high initial investment required, additive manufacturing has the potential to significantly reduce costs in mass production. Once a suitable digital production process and optimized supply chain management have been established, additive manufacturing can enhance production efficiency, reduce intermediate processes and inventory costs, and thus become cost-effective over time. The significant benefits of metal additive manufacturing technology have led to its widespread adoption in a number of industrial sectors, including aerospace [26], automotive [27], biomedical [28,29], construction [30], and others [31,32].

Many studies have demonstrated the various distinctive advantages of metal AM technology. However, some limitations remain, such as the anisotropy in microstructure and mechanical properties [1,33,34,35,36]. Anisotropy describes the various orientation characteristics of a material. Numerous studies have demonstrated that metal AM parts exhibit some degrees of anisotropy in microstructure and mechanical properties. In traditional practice, metal AM parts’ excellent and stable mechanical properties are a prerequisite for engineering applications [37,38,39]. Researchers have studied the microstructures, mechanical properties, and processing characteristics of various metals and alloys in different metal AM systems. Among these, the formation of anisotropy and its impact on mechanical properties have emerged as a significant research topic in metal AM.

In contrast to metal components manufactured using traditional techniques, metal AM components typically undergo more intricate thermophysical processes during forming [23,40,41], resulting in the formed parts’ microstructure and mechanical properties exhibiting anisotropy. Consequently, it is ubiquitous to observe anisotropy in metal AM components. To obtain a deeper insight into the thermodynamic processes involved in metal AM forming and the microstructure and properties of the formed components, we present a review of the literature on the thermodynamic processes involved in metal AM manufacturing and the anisotropy of the microstructure and mechanical properties of different types of metal parts after forming.

## 2. Thermal-Affected Zone (Thermophysical Processes of Additive Manufacturing Metal Materials)

**Figure 1:**
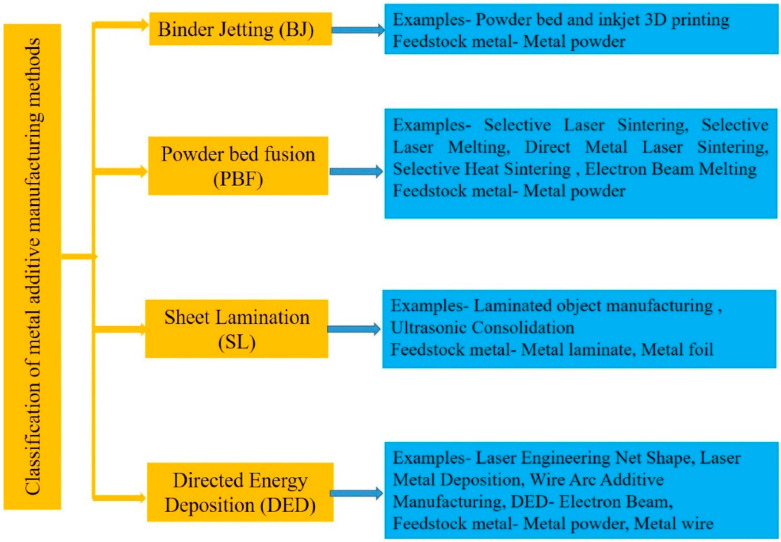
Typical types of metal AM techniques (reproduced with permission from Ref. [44]. Copyright (2023), Elsevier).

As illustrated in Figure 1, binder jetting (BJ), powder bed fusion (PBF), sheet lamination (SL), and directed energy deposition (DED) are prevalent in AM technologies for the fabrication of components utilizing metal materials. However, practical metalworking uses PBF and DED more commonly than BJ and SL. Two key factors contribute to the prevalence of PBF and DED. Firstly, PBF, DED, and BJ can use powdered metal materials to manufacture products, whereas SL is limited to using sheet metal as a raw material. Generally, specimens manufactured using powder as raw materials are more accurate and have a better surface finish. Conversely, specimens manufactured using wire and sheet metal as raw materials are more prone to defects, lower geometrical accuracy, higher surface roughness, and limited ability to fabricate complex-shaped specimens [42,43].

PBF and DED techniques have also become the dominant techniques for preparing high-performance metal AM specimens because they can produce near-net-shape components directly from computer models rather than requiring additional steps to achieve the designed shape. This section will also provide an overview of the PBF and DED technologies and the material’s microstructure during molding.

### 2.1. Powder Bed Fusion

**Figure 2:**
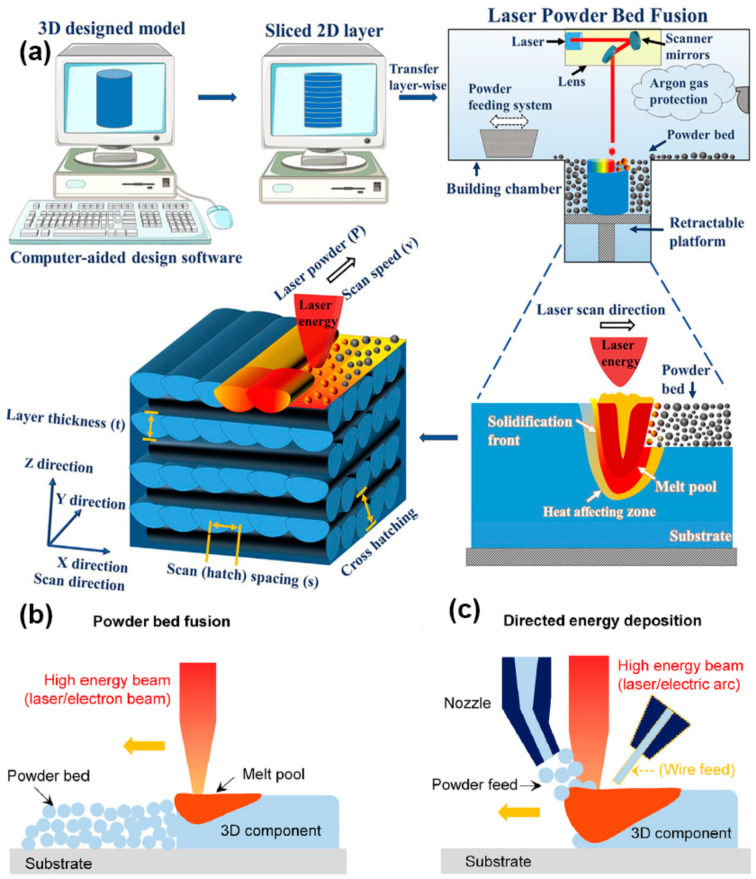
Schematic diagram of additive manufacturing: (**a**) process of AM technology (reproduced with permission from Ref. [46]. Copyright (2023), Elsevier); (**b**) PBF; (**c**) DED (reproduced with permission from Ref. [2]. Copyright (2022), Elsevier).

**Figure 3:**
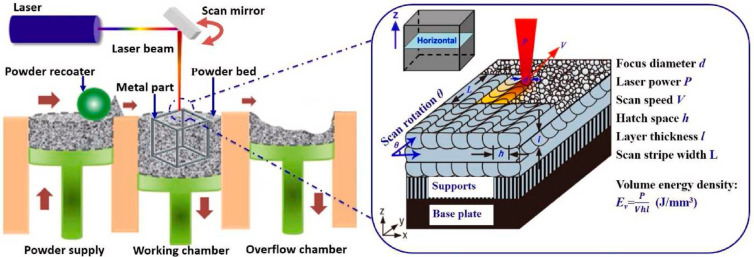
Illustrations of the laser PBF technology (reproduced with permission from Refs. [47,48]. Copyright (2017), (2015) Elsevier).

As shown in Figure 2, the preparer first models the object using computer-aided design (CAD, Version: R.47.0.0 AuoCAD 2021) software. The printer then divides the three-dimensional model into layered sections and prints the material layer by layer. Three-dimensional printing is performed by depositing single layers and locally melting the material with a heat source to create the three-dimensional physical component. In the L-PBF (laser-PBF) process shown in Figure 3, the main process parameters include the spot diameter (d), laser power (P), scan speed (V), hatch space (h), layer thickness (l), scan stripe width (L), and laser scanning strategy. The volume energy density (*E_V_*) when the laser interacts with the powder is often calculated using Equation (1) [45]:

(1) $$E_{v}=\frac{P}{Vhl}$$

The smaller size of the laser spot enables products manufactured using PBF to exhibit a greater surface finish and greater accuracy. Secondly, PBF technology can be manufactured from a wider variety of engineered materials, thereby increasing its versatility. Finally, it enables the fabrication of components with intricate geometrical configurations. Furthermore, when coupled with topology optimization, the technique enables the fabrication of lightweight metal components from reduced material volumes, a capability that is pivotal in sectors such as automotive and aerospace. Concurrently, the PBF process is not without its shortcomings. The necessity for high part density and a high cooling rate can result in alterations to the microstructure of the manufactured part. Consequently, parts manufactured by PBF typically exhibit poor ductility. Furthermore, other defects, such as spheroidization, and porosity, can also affect the LPBF process, particularly in terms of fatigue performance. The applications of high cooling rates and temperature gradients result in the formation of residual stresses, which have a significant impact on the initiation of cracks.

### 2.2. Directed Energy Deposition

**Figure 4:**
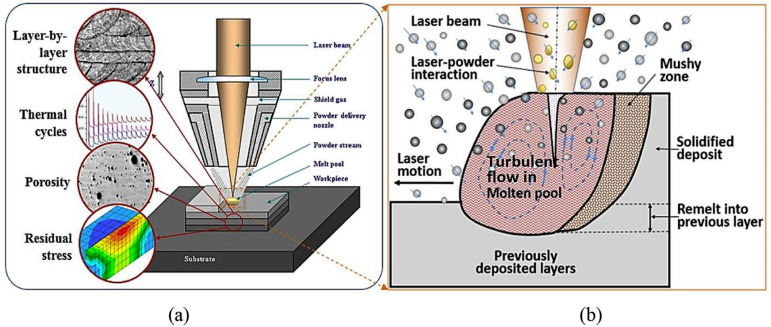
Schematic diagram of DED (**a**) microstructure, multiple interfaces, thermocycles, defects, and residual stress, and (**b**) interaction among injected powder, laser beam, and melt pool. (Reproduced with permission from Ref. [49]. Copyright (2019), Elsevier).

DED AM is a method of forming and manufacturing parts using a high-energy laser beam to melt powder/wire material through a “discrete + stacking” method based on the three-dimensional data model of the part under computer control. As illustrated in Figure 2, this technology employs a high-power laser as a moving heat source to feed metal powder/wire material into a melt pool, which then solidifies and cools on the substrate. DED path planning is analogous to L-PBF, whereby the laser deposits layers to obtain three-dimensional metal parts. Figure 4 illustrates that this technology involves several process parameters, including laser power (P), scan speed (V), hatch space (h), powder feed rate, shield gas flow rate, and flow rate of powder carrier gas.

DED technology can produce large near-net-shape freeform components and is widely used to repair high-value components in the aerospace, biomedical, and automotive industries. Therefore, DED is one of the most cost-effective and versatile laser AM methods. Compared with L-PBF technology, DED technology achieves higher manufacturing efficiency using higher laser power and larger laser beam sizes. In addition, DED is also suitable for the manufacture of gradient structures with multiple materials fed synchronously and for the repair of high-performance and high-value components. However, it is not easy to manufacture parts with highly complex geometries using DED technology. Moreover, rapid cooling during the DED process can lead to defects in the formed parts, including significant residual stresses and poor microstructural characteristics, such as porosity, cracks, or large epitaxial grains. These issues limit the widespread industrial application of DED in producing safety-critical components.

### 2.3. The Melt Pool in Metal Additive Manufacturing

**Figure 5:**
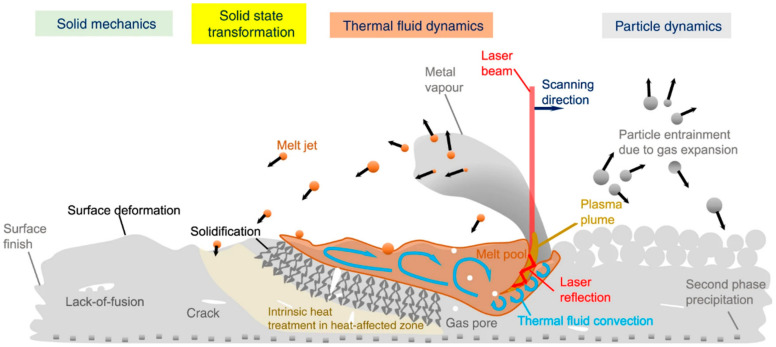
A schematic illustration of multi-scale, multi-physics phenomena in PBF AM. (Reproduced with permission from Ref. [41]. Copyright (2020), Springer Nature).

The unique layer-by-layer deposition process in AM exposes the product to complex thermophysical dynamics, as shown in the AM process schematic and illustrated in Figure 5 [41]. Therefore, researchers have utilized the unique cyclic heat treatment effects in laser AM to control the microstructure of formed parts (such as precipitated phases) and improve the comprehensive mechanical properties of materials. The complex thermophysical processes in AM also allow researchers to develop new materials specifically for laser AM. For example, Bright Laser Technologies Co., Ltd., Xi’an, China has independently developed more than 10 types of special powder materials [50], such as TAM1, AIAM1, TC18, In738, K452, etc., which solves the problems of high residual stress in the forming and deposition states of the traditional grades of materials, poor adaptability of the process, and cracking in printing, and avoids the emergence of cracking and deformation in the process of 3D printing. Due to the variety of printers, the differences in materials, and the need for fine-tuning a range of parameters, establishing a connection between them remains challenging. It is well known that the service performance of materials is closely related to internal defects; if these defects cannot be effectively eliminated or suppressed, they can lead to premature structural failure and serious accidents. Since metal materials are not easily affected by cracking mechanisms originating from the liquid phase (such as liquation cracking or hot tearing) and are also not easily affected by solid-state stresses (such as strain-age cracking and ductility dip cracking), the candidate materials for metal AM are often weldable alloys. Metal AM fundamentally involves a complex physical process that couples solid, liquid, and gas phases [41]. During forming, materials are often prone to structural defects, such as pores and cracks, due to solidification shrinkage and thermal stresses [45]. Therefore, understanding the formation mechanisms of defects during the metal AM process and providing relevant recommendations are of great significance for material preparation. Additionally, the service performance of materials is related to their microstructural morphology, which closely links to the processing parameters of the forming. How to select appropriate process parameters among numerous combinations and clarify the influence of these parameters on microstructural morphology is a primary research focus in the field of metal AM technology. Thus, researchers must study the forming processes and materials’ service performance and microstructure.

**Figure 6:**
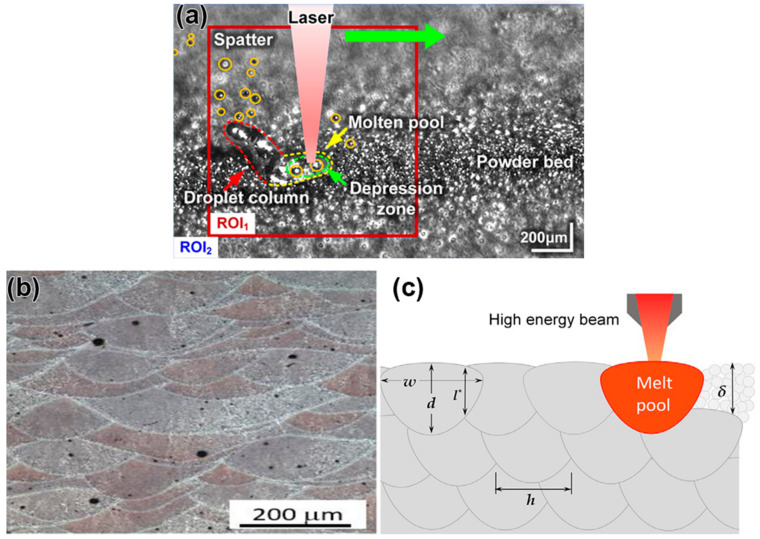
(**a**) Melt pool of the laser–matter interaction during the L-PBF of an Inconel 718 powder bed (reproduced with permission from Ref. [52]. Copyright (2019), Elsevier), (**b**) scanning electron microscope image of Al alloy melt pool (reproduced with permission from Ref. [53]. Copyright (2020), Elsevier), and (**c**) schematic diagram of melt pool (reproduced with permission from Ref. [2]. Copyright (2022), Elsevier).

The melt pool (Figure 6) is the smallest fundamental unit in the processing and forming of laser AM. The stability of the melt pool is the guarantee for the stability of the entire processing process and even the final formed part’s organizational performance. Analyzing the changes in the morphology characteristics of the melt pool in different process forming processes plays an essential role in optimizing the performance of the formed parts. The size of the melt pool is related to the parameters of the energy sources used in the AM process, such as laser, electron beam, and arc. The morphology of the melt pool and the solidification mode directly affect the performance and quality of the final component. Thus, fine control of the melt pool’s morphology and solidification process is required.

**Figure 7:**
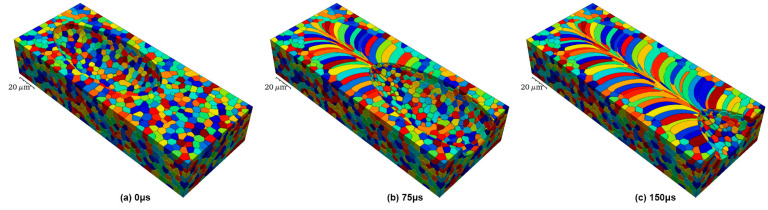
Predicted microstructural evolution of the melt pool: (**a**) 0 µs (initial condition), (**b**) 75 µs, and (**c**) 150 µs (reproduced with permission from Ref. [51]. Copyright (2021), Elsevier).

The melt pool refers to a localized high-temperature zone caused by the concentration of energy from the laser beam on the material surface or depth, where the material melts into a liquid state. When the laser beam acts on the material surface, the heated area is small, and the melt pool depth is relatively small; when the laser beam penetrates the material, the melt pool depth and diameter are relatively large. Figure 7 illustrates the evolution of the predicted microstructure of the melt pool at three time intervals: (a) 0 µs (initial condition), (b) 75 µs, and (c) 150 µs. As time progresses, the laser advances along its trajectory, causing grains to melt along the leading edge of the melt pool. It is evident that the grains that contact the melt pool at its widest point are not necessarily completely melted. As the melt pool continues to advance, these grains begin to solidify along the trailing edge of the melt pool. In general, the formation of grains with much higher aspect ratios than the initial microstructure is observed, with these grains bending from the sides of the bath toward the center of the laser track. The formation and development of the molten pool is divided into four stages. The first stage is the heat transfer process, where there is a transient heat transfer between the laser beam and the material. The laser beam is focused on the surface or depth of the material, creating a small area of high temperature. The material within this region heats up instantaneously, while the surrounding material gradually warms up through heat transfer, leading to a phase transition. As the surface temperature of the material increases, the surface tension Coulomb force becomes greater and a layer of liquid metal begins to form on the surface of the material. The second stage is the formation of a liquid melt pool. Since the temperature of the instantaneous heat transfer is highly consistent with the melting point of the material, the material melts rapidly after the instantaneous heating. Over time, the depth and temperature of the material heated by the laser beam increase, resulting in the formation of a molten pool. The formation of the melt pool is due to the plastic deformation of the material in a high-temperature environment. The shape and size of the molten pool are determined by factors such as laser processing parameters, metal powder properties, material melting point, and surface tension [51]. The third stage is melt pool diffusion. After the formation of the melt pool, the temperature and pressure inside the melt pool are constantly changing, resulting in the flow and diffusion of the material, which eventually forms the desired solid shape. The temperature gradient and concentration gradient in the melt pool determine the complex shape of the material formed in the melt pool. In addition, factors such as melt pool hydrodynamics, mass transport, and the rate at which the melt pool encounters cooling also influence melt pool diffusion. The last stage is the solidification stage of the molten pool, where the material melts in the molten pool and solidifies rapidly to form a solidified portion. Due to the inhomogeneity of the material in the rapid cooling process, the structure and nature of the solidified part will also change. In short, the formation and evolution of the melt pool are some of the core processes of AM. Understanding and optimizing the morphology and temperature gradient of the melt pool can improve manufacturing quality and productivity.

#### Heat-Affected Zone in Metal Additive Manufacturing

**Figure 8:**
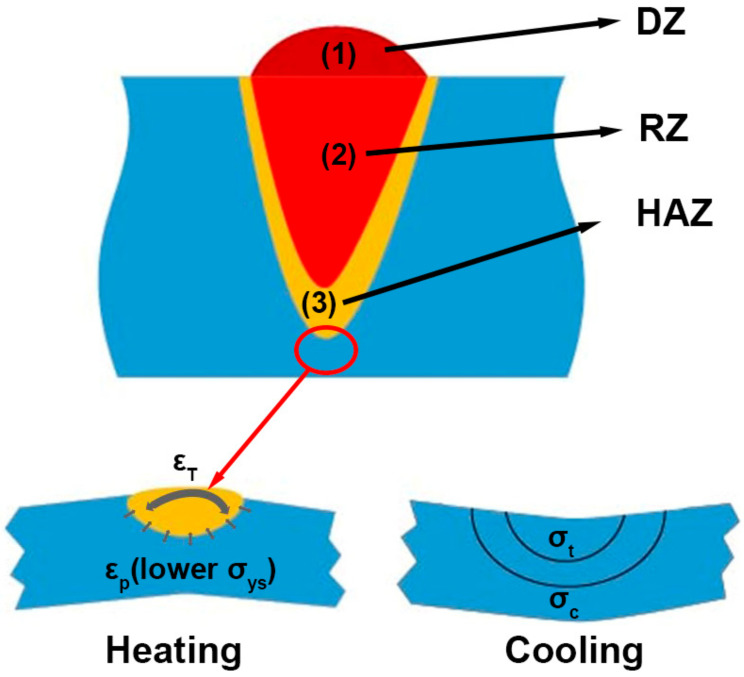
Schematic diagram of the melt pool’s composition and residual stress generation.

The rapid melting and solidification of the AM process results in significant temperature gradients, leading to thermal stresses that cause defects, such as deformation and cracking. As shown in Figure 8, the melt pool is typically composed of three regions: the deposition zone (DZ) at the top, the remelting zone (RZ) at the bottom, and the heat-affected zone (HAZ). During the AM process, the powder undergoes rapid heating, instantaneous melting, and rapid solidification in each layer-forming process. The high-temperature melt pool formed by the melting of the powder will generate heat input to the already solidified material at the bottom through heat conduction and radiation. The heat input acting on this layer will reappear in the subsequent layers’ deposition process, resulting in the periodic thermal treatment of the already solidified material. When the already solidified material undergoes periodic thermal treatment, it absorbs the generated heat, forming a region between the molten metal and the already solidified metal, the heat-affected zone. In laser AM, a heat-affected zone still exists despite the concentration of heat source energy density and the relatively small HAZ. Addressing the evolution of material properties within this zone remains a critical issue. As shown in Figure 8, when the laser beam irradiates the material in the selected area, the material rapidly melts and forms a melt pool. During the heating process, the material in the melting zone is in a complete plastic deformation state, and there is no stress at this stage. The heat-affected zone expands due to heating but is restricted by the unaffected zone, resulting in compressive stress in the heat-affected zone and tensile stress in the unaffected zone. When the laser beam stops irradiating or leaves this area, the material in this area enters the cooling process. The material in the melting zone changes from a state of complete plastic deformation to an incomplete plastic state, and its volume shrinks due to cooling. However, the restriction of the HAZ generates tensile stress in the melting zone and compressive stress in the heat-affected zone. Currently, there is still some tensile stress in the unaffected zone. When the material in this area is completely cooled, there is tensile stress in the original melting zone, while compressive stress is present in the original heat-affected and unaffected zones. In addition to the stresses introduced by the laser selective melting process, the phase transformation of the material can also introduce some stresses. Therefore, it is necessary to preheat the substrate before manufacturing. A preheated substrate can effectively suppress crack formation by reducing the temperature gradient between the high-temperature melt pool and the solidification zone, forming a more uniform temperature field around the melt pool, reducing the heat dissipation rate, and thus reducing the residual stress of the formed part. Moreover, preheating the substrate reduces the yield strength (YS) of the material, allowing the material to undergo plastic deformation at lower equivalent stresses, which is beneficial for releasing residual stresses and improving the material’s mechanical properties.

As previously mentioned, thermal cycling input from the high-temperature melt pool formed by powder melting creates the HAZ. The thermal cycling affects the solidified material at the bottom through heat conduction and radiation. Combined with Formula (1), the factors affecting the cyclic thermal input in the forming process include process parameters, deposition direction, interlayer delay time, substrate preheating, and laser remelting. Process parameters affecting cyclic thermal input include laser power, scanning speed, scan spacing, and scanning strategy. Deposition direction, interlayer delay, and laser remelting also play auxiliary roles in controlling the size of the cyclic thermal input. Substrate preheating mainly acts as an external heat source coupled with the cyclic thermal input in the temperature field. By controlling the above parameters, the behavior of cyclic thermal input can be fully regulated, thereby regulating the microstructure evolution and mechanical performance of laser AM deposited materials [54,55]. Additionally, the width of the HAZ is closely related to the material’s thermal diffusivity, which determines its size. It is the thermal conductivity of the metal under constant pressure divided by the ratio of its density and specific heat capacity. In summary, the thermal diffusivity of metal measures the speed at which heat transmits through its body. If the thermal diffusivity is high, the metal can transmit heat more quickly, resulting in a larger HAZ. For example, the thermal diffusivity of AISI304 stainless steel is 4.2 mm^2^/s, while structural steel’s is 11.72 mm^2^/s. The considerable difference in thermal diffusively indicates that, compared with AISI304 stainless steel, structural steel has a much broader HAZ when heated. The HAZ’s generation also depends on various other factors, and the width of the area depends on the amount of heat generated, the duration of exposure to heat, and the thickness of the material. Thinner metal sheets heat up more quickly, thus forming a larger HAZ.

The size of the HAZ increases with rising laser power. High-power lasers create larger melt pools on the substrate, prolonging the solidification and cooling time of the molten metal. A larger melt pool requires more prolonged cooling, resulting in a larger HAZ. On the other hand, low laser power produces smaller melt pools. The smaller melt pool solidifies quickly and does not allow too much heat to pass through the substrate, resulting in a smaller heat-affected zone.

The thermal cycling in laser AM can cause changes in the material’s microstructure, ultimately affecting its properties. The thermal process can influence grain size and uniformity, the types, distribution, and size of precipitated phases, solid solubility, and the degree of element segregation at grain boundaries and other factors. These factors collectively impact the HAZ’s hardness, strength, ductility, corrosion resistance, and other properties. The incomplete recrystallization zone, the recrystallization zone, the overheating zone, and other subzones form a typical HAZ from the base metal to the interface. The incomplete recrystallization zone has poor grain size uniformity and performance uniformity; the recrystallization zone usually has a finer structure; and the overheating zone has many abnormally grown grains, and its grain size and performance uniformity are also poor. Metal materials commonly used in AM include titanium alloys, aluminum alloys, stainless steel, magnesium alloys, high-temperature alloys, and others. Therefore, the degradation of the HAZ’s performance in laser AM also varies greatly depending on the specific material, requiring targeted exploratory research. In welding processes, there is also an HAZ: the area where the base metal changes its metallographic structure and mechanical properties due to heat but without melting. Compared with the HAZ produced in welding processes, the HAZ in AM has more significant temperature gradients, more drastic temperature changes, potentially more cycles of thermal cycling, and thus more complex microstructures and properties. Therefore, studying the effects of cyclic thermal input on materials and microstructure properties is crucial for improving AM quality and developing specialized materials for AM. Therefore, a thorough summary and study of the HAZ generated in AM is necessary.

## 3. Additive Manufacturing-Fabricated Alloys and Their Anisotropy

Since the inception of AM technology for metal components, its development has closely intertwined with advancements in complementary materials. In recent years, there has been significant interest in the specialized metal powder materials used in the AM of metal components, such as titanium alloys, high-temperature alloys (nickel-based, cobalt-based, and iron-based), stainless steel, aluminum alloys, magnesium alloys, and other alloys. Some countries have already achieved laser direct forming of small stainless steel, high-temperature alloys, and other parts. In the future, laser rapid forming of large metal components of high-temperature and titanium alloys will become the main focus of technological breakthroughs [56].

Metal components manufactured using AM technology can address issues associated with traditional manufacturing methods, such as high costs, low material utilization, complex processes, and challenging post-processing. However, researchers have found that the process and parameters of AM lead to anisotropy in the microstructure and mechanical properties of the final components [35,40,57,58,59]. In established engineering practices, the exceptional and reliable mechanical properties of metal components produced through AM are highly regarded. Therefore, traditional applications rarely use functionally graded materials (FGMs) with anisotropic properties. As a result, researchers are more concerned with eliminating the anisotropy of metal components manufactured using AM through post-heat treatment [58,60,61,62]. Since Niino et al. first proposed manufacturing thermal gradient metal–ceramic phases for thermal barrier applications, there has been a deeper understanding and appreciation for functionally graded materials (FGMs) [63]. Compared with isotropic bulk materials, the composition and structure of FGMs can be precisely designed for customized multifunctional properties. Therefore, FGMs have broad applications in aerospace engineering, nuclear power generation, sensors, biomedical implants, optoelectronic devices, energy absorption systems, and other fields. Subsequently, anisotropy formation in metal materials manufactured using AM and its impact on mechanical properties have become a hot research topic. This section will review several typical applications of metal materials produced using AM technologies and the anisotropy of their microstructure and properties.

**Figure 9:**
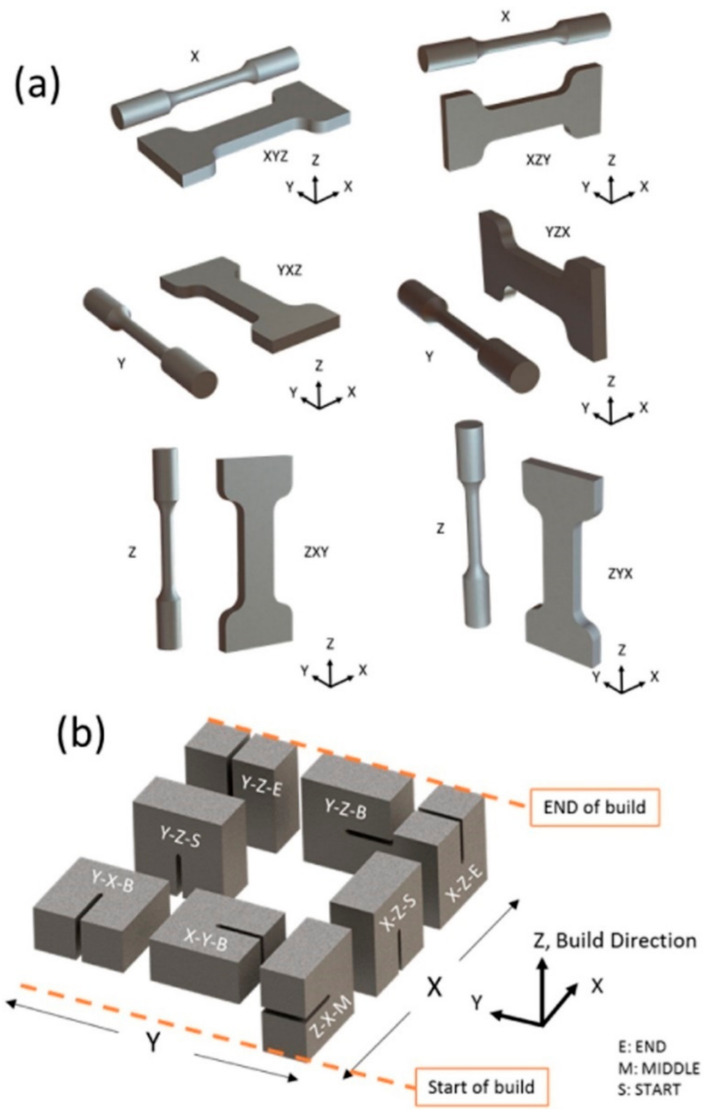
ISO and ASTM standards for orientation designations for AM (**a**) tensile test, and (**b**) ISO designation for the orientation dependence of mechanical properties of AM-processed samples (reproduced with permission from Ref. [35]. Copyright (2018), Elsevier).

Table 1 provides statistics on the mechanical tensile properties of additively manufactured metals using direction codes for mechanically testing specified in ISO and ASTM standards for consistency in comparison. To standardize testing standards for AM [64], ISO/ASTM jointly developed a set of AM standards used globally, with the first set of standards was released in 2013. Figure 9a shows the direction codes for mechanical testing based on ISO and ASTM standards. In this terminology, specifying the direction for tensile testing requires three letters: X, Y, and Z. The X-axis is specified as parallel to the machine front, while the Z-axis is in the vertical direction. Following the right-hand rule coordinate system, the Y-axis is perpendicular to the X and Z axes. The first letter in the name corresponds to the axis parallel to the longest overall dimension, and the second and third letters correspond to the axes parallel to the second and third longest overall dimensions, respectively.

Furthermore, Figure 9b illustrates the ISO terminology for the orientation correlation of mechanical properties of AM machined samples, as determined by the study of Seifi et al. [65]. In this terminology, the first letter denotes the orientation of the crack plane, the second letter denotes the predicted direction of crack extension, and the last letter denotes the plane that creates the crack. For instance, the designation Y-X-B signifies that the normal direction of the crack plane is aligned with the Y-axis, the direction of crack propagation is along the X-axis, and the crack originates at both ends.

**Table 1:** Mechanical tensile properties of alloys fabricated by AM techniques with different tensile axes at room temperature.

Materials	Method	Condition	Tensile Axis Orientation	σ_0.2_ (MPa)	σ_UTS_ (MPa)	ε (%)	Refs.
Ti6Al4V	L-PBF	As-built	Horizontal	1075 ± 25	1199 ± 49	7.6 ± 0.5	[66]
			Vertical	967 ± 10	1117 ± 3	8.9 ± 0.4	[66]
Ti6Al4V	L-PBF	As-built	Horizontal	1070 ± 50	1250 ± 50	5.5 ± 1	[59]
			Vertical	1050 ± 40	1180 ± 30	8.5 ± 1.5	[59]
Ti6Al4V	EB-PBF	Machined	Horizontal	1063	-	7.1	[67]
			Vertical	997	-	8.8	[67]
Ti6Al4V	EB-PBF	Machined	Horizontal	817 ± 6	916 ± 13	9.3 ± 1.6	[40]
			Vertical	835 ± 15	949 ± 4	18.2 ± 0.7	[40]
Ti6Al4V	EB-PBF	As-built	Horizontal	870 ± 8.1	971 ± 3.1	12.1 ± 0.8	[68]
			Vertical	879 ± 12	953 ± 8.8	13.8 ± 0.9	[68]
Ti6Al4V	DED	As-built	Horizontal	892 ± 10	911 ± 10	6.4 ± 0.6	[69]
			Vertical	522	797 ± 27	1.7 ± 0.3	[69]
Ti6Al4V	DED	Machined	Horizontal	960 ± 26	1063 ± 20	10.9 ± 1.4	[34]
			Vertical	958 ± 19	1064 ± 26	14 ± 1	[34]
Ti6Al4V	DED	Machined	Horizontal	1027 ± 6	1077 ± 14	2.9 ± 0.6	[70]
			Vertical	1031 ± 68	1106 ± 52	6.8 ± 1.2	[70]
CP-Ti (Grade2)	L-PBF	As-built	Horizontal	533 ± 2.1	617 ± 16.7	5.1 ± 2.1	[71]
			Vertical	522 ± 18	654 ± 15	17 ± 3	[71]
316L stainless steel	L-PBF	As-built	Horizontal	528 ± 4	659 ± 3	16.6 ± 0.4	[72]
			Vertical	444 ± 27	567 ± 19	8 ± 2.9	[72]
304L stainless steel	L-PBF	As-built	Horizontal	568 ± 2	715.5 ± 1.5	41.7 ± 1.1	[73]
			Vertical	450	550	57	[73]
Al-Si-10Mg	L-PBF	As built	Horizontal	169 ± 1	272.8 ± 2.9	8.2 ± 0.3	[74]
			Vertical	168.8 ± 1.3	267	9.1 ± 0.5	[74]
Al-12Si	L-PBF	As built	Horizontal	270.1 ± 10	325 ± 20	4.4 ± 0.7	
			Vertical	274.8 ± 8	296.1 ± 20	2.2 ± 0.3	
Al-12Si	L-PBF	HT	Horizontal	153.4 ± 5	228 ± 13	5.3 ± 0.7	[75]
			Vertical	150.3 ± 17	210.1 ± 20	4.2 ± 0.3	[75]
IN718	L-PBF	-	Horizontal	816 ± 24	1085 ± 11	19.1 ± 0.7	[76]
			Vertical	737 ± 4	1010 ± 10	20.6 ± 2.1	[76]
IN718	L-PBF	HT	Horizontal	1222 ± 26	1417 ± 4	15.9 ± 1	[76]
			Vertical	1186 ± 23	1387 ± 12	17.4 ± 0.4	[76]

HT = heat-treated. UTS = ultimate tensile strength. ε represents elongation.

### 3.1. Additive Manufacturing-Fabricated Ti and Ti Alloys

Titanium and titanium alloys are AM’s most commonly used metal materials [71,77]. Due to their advantages, such as high-temperature resistance, high corrosion resistance, high strength, low density, and biocompatibility [78], titanium alloys have been widely used in the aerospace industry [56,79], sports equipment [23,80], and medical device [81,82] fields.

High-tech fields widely use titanium alloy components that traditional forging and casting techniques produce. However, producing large titanium and titanium alloy parts using traditional forging and casting methods face many challenges, such as high product costs, complex processes, low material utilization rates, and difficulties in subsequent processing [78]. These issues have hindered titanium and titanium alloys’ broader application and research. Compared with traditional forging and casting methods, AM technology can fundamentally solve these problems. Therefore, this technology has become a new technology for directly manufacturing titanium alloy parts in recent years. Since the 1960s, clinicians have successfully used additively manufactured titanium alloy implants as biomaterials for medical implants in the spine, hips, knees, and limbs. AM uses materials such as commercially pure titanium (CP-Ti), Ti-24Nb-4Zr-8 (Ti2448), Ti6Al4V (TC4), Ti-6Al-7Nb,Ti-42Nb [83,84,85,86], and Ti-15Mo-5Zr-3Al [87,88,89,90], which have good biocompatibility and high mechanical strength, making them widely used in medical implants [77]. Among all commonly used implant materials, titanium has good biocompatibility and mechanical properties, is non-magnetic, and its density and elastic modulus are close to that of human bone, thereby avoiding the ’stress-shielding’ effect [91,92]. In addition, AM technology, with its advanced characteristics combining mechanical, computational, CNC, and material properties, enables the customization of porous structures for bone implants (thus achieving bone integration) and better patient treatment outcomes. In the past, people with rare diseases often could not receive effective treatment because of their uniqueness. However, with AM technology, implants can be produced specifically designed for individual patients. Therefore, among stainless steel, cobalt–chromium–molybdenum alloys, and titanium, titanium is the most promising biomaterial for biomedical applications.

In comparison to the higher Young’s modulus of α-type Ti-6Al-4V additively fabricated β-type titanium alloys, such as Ti-42Nb, Ti-15Mo-5Zr-3Al, etc., β-type titanium alloys provide a favorable basis for use as bio-implants due to their good biocompatibility and unique low Young’s modulus. The Young’s modulus of cast β-titanium alloys remains elevated in comparison to cortical bone, which results in a stress-shielding effect [93,94,95]. The implant assumes the applied stresses instead of the bone, thereby inhibiting bone regeneration and simultaneously increasing bone resorption by the body. Consequently, complications such as implant loosening or bone weakening may ensue. As outlined in the literature, a novel approach to reducing the elastic modulus of biocompatible β-type titanium implants is to control the crystallographic orientation distribution of the grains (crystallographic weaving). The literature indicates that the Young’s modulus of β-titanium alloys is anisotropic, with the modulus of elasticity varying with different orientations. Ishimoto [87] et al. prepared two distinct textures of beta-type Ti-15Mo-5Zr-3Al materials via the L-PBF technique. In this work, the authors were able to control the growth of columnar cells in the molten pool by means of a scanning strategy, which allowed the production of these different textures. The obtained material has a low Young’s modulus of 68.7 ± 0.9 GPa, which could be used for the development of stress-shielding implants. Pilz [83] et al. also used laser control to prepare Ti-42Nb with a weave parallel to the build direction <0 0 1>, and obtained a material with a Young’s modulus as low as 44 GPa, which represents a significant reduction of more than 30% when compared to that of the Gaussian reference sample (67–69 GPa). In addition to biomedical implants, the process of using the anisotropy of additively manufactured metal materials to produce adaptable low Young’s modulus molded parts has significant potential for aerospace and biomedical applications.

The outstanding characteristics of titanium alloys, including low density, high specific strength, low thermal conductivity, good high- and low-temperature performance, and strong corrosion resistance, make them indispensable structural materials in many industrial sectors [96]. High-tech fields, such as aerospace, first used titanium alloys. The proportions of titanium alloys used in U.S. military aircrafts, such as F14, F15, F117, F22, and B2, are 24%, 27%, 25%, 26%, and 42%, respectively. The amount of titanium alloy used in a Boeing 747 aircraft is 42.7 tonnes [56,97]. The widespread use of titanium alloys has earned them the nickname ’space metals’. The application and use of high-end titanium alloy materials also reflect a country’s level of aerospace technology development. Titanium alloys are also known as ’marine metals’. Due to their excellent corrosion resistance, they are not affected by seawater corrosion. Additionally, titanium alloys are high-quality, lightweight structural materials widely used in marine engineering. The 21st century is known as the ocean century, and accelerating the development of marine high-tech has become an essential strategic deployment for coastal countries worldwide. Titanium metal is a critical material in the field of marine engineering. With the development of national marine construction, the application of high-end titanium materials in marine engineering will increase significantly. Furthermore, titanium is also widely used in the automotive and chemical industries and daily life [96].

### 3.2. Anisotropy in Additive Manufacturing-Fabricated Ti and Ti Alloys

Ti6Al4V (TC4) is the most widely researched titanium alloy in AM because it accounts for more than 50% of total titanium usage [78]. Researchers manufactured a Ti6Al4V (TC4) alloy using various AM techniques to evaluate the performance of each process. It was also one of the first alloys used in industrial production with selective laser melting (SLM). Many researchers have studied the mechanical properties of Ti6Al4V alloys formed using SLM technology. Many studies have also shown that Ti6Al4V alloys manufactured by AM technology can achieve mechanical properties comparable to traditional casting and forging materials [57,69,78]. Despite AM’s simplicity and speed advantages, researchers pay less attention to the anisotropy of titanium alloys produced using different AM technologies. More importantly, whether the performance of metals manufactured in the worst direction by AM can meet the minimum requirements of traditional casting and forging metals remains unclear. Therefore, research on the anisotropy of AM materials is necessary. Simonelli [66] and Qiu et al. [58] found that build direction influences the tensile properties of Ti6Al4V alloys formed by SLM, especially the ductility. Vialro et al. [98] found that the mechanical properties of SLM-formed Ti6Al4V specimens, especially ductility, exhibit anisotropy due to defects in different directions. Based on previous studies, the authors summarized the tensile properties of metals manufactured using different AM technologies in different directions, as shown in Table 1. Figure 4 shows the schematic diagram of the tensile property test directions in the table.

**Figure 10:**
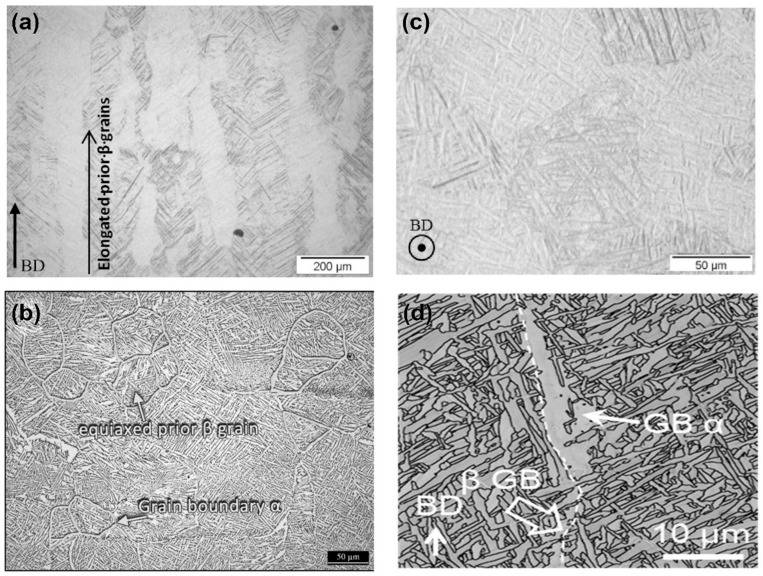
Micrographs of the macrostructure of Ti alloy: (**a**) side view parallel to the building direction (BD) of Ti6Al4V SLM (reproduced with permission from Ref. [102]. Copyright (2022), MDPI), (**b**) equiaxed grains and grain boundary α of Ti-6Al-4V–7Cu sample (reproduced with permission from Ref. [105]. Copyright (2022), Elsevier), (**c**) top view perpendicular to the BD of Ti-6Al-4V SLM (reproduced with permission from Ref. [102]. Copyright (2022), MDPI), and (**d**) EBSD α grain boundary (reproduced with permission from Ref. [100]. Copyright (2021), Elsevier).

Table 1 demonstrates that titanium alloys produced using the same AM technology exhibit unique mechanical property differences between the perpendicular (horizontal) and parallel (vertical) directions to the build direction, attributable to the microstructure of the metal AM-formed part [99]. As shown in Figure 10, in the side view of the SLM sample (Figure 10a), vertical columnar prior-β grains are observed. Long columnar prior-β grains grow along the BD direction, which results from the remelting of the previously solidified layer during the SLM process. Additionally, needle-like α′ martensite forms inside the prior-β grains in the top view of the SLM sample (Figure 10c) because of the high cooling rate of the L-PBF process. Meanwhile, due to the HAZ, the α phase may also exist in the sample due to slower cooling rates [100] (Figure 10d, more common in DED processes). In the L-PBF process, the direction of the temperature gradient relates to the formation of columnar grains. The crystal growth rate is much higher when the crystal growth direction is consistent with the maximum temperature gradient. Therefore, the grains will grow toward the melt pool along the BD direction [101]. Titanium alloy components formed using L-PBF, electron beam PBF (EB-PBF), and DED technologies commonly contain columnar prior-βand equiaxed grains [40]. Columnar prior-β grains are more common than equiaxed grains, and they tend to grow along the temperature gradient direction and extend across multiple layers [45,102]. On the boundaries of columnar prior-β grains, grain boundary α′ phases exist. In comparison to titanium alloy components produced by L-PBF and DED methods, the grain boundary α′ grains in titanium alloy components fabricated by EB-PBF technology are observed to be somewhat smaller [40]. The existing literature indicates that the formation of the acicular martensitic α′ phase in Ti-6Al-4V alloy processed by L-PBF is influenced by three key factors: the cooling rate, the formation of scan tracks, and epitaxial grain growth [45,103]. For Ti-6Al-4V components formed by traditional casting and forging processes, the presence of the grain boundary α′ phase along the prior-β grain boundaries provides a preferential path for damage accumulation, thereby reducing their elongation rate [104].

**Figure 11:**
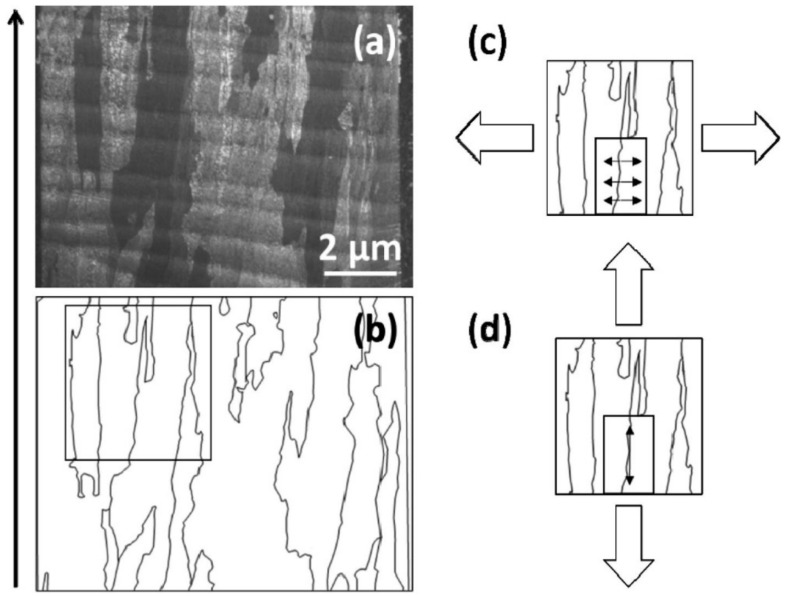
(**a**) Micrograph showing build lines and longitudinal direction oriented horizontally, and prior-β grains and transverse direction oriented vertically; arrow indicates build direction. (**b**) Outline of prior-β grain boundaries from micrograph in (**a**), along which grain boundary α phase is present as shown in this figure. (**c**,**d**) The illustration depicts the schematic representation of the forces acting on the microstructure within the box in (**b**) when subjected to tensile stresses in varying directions. (Reproduced with permission from Ref. [34]. Copyright (2015), Elsevier).

As shown in Figure 11, the reasons contribute to the anisotropy in the ductility of titanium alloy components prepared using AM technology. Firstly, when we apply tension parallel to the short axis of the prior-β grains, both the short axis of the prior-β grains and some grain boundary α′ grains are stretched. In this case, only the grain boundary α′ grains and some prior-β grain boundaries bear the load, leading to a reduced effective slip distance between adjacent grains. Conversely, when tension is applied parallel to the long axis of the prior-β grains, the long axis of the prior-β grains and the entire grain boundary α′ grains are subjected to tension, resulting in an increased effective slip distance between adjacent grains. Therefore, the anisotropic microstructure causes the elongation rate of the component to be greater, parallel to the build direction than perpendicular to it. Additionally, this anisotropic microstructure is the fundamental reason for the strength differences in different directions of the component. As mentioned earlier, due to the temperature gradient during the forming process of titanium alloy components, the columnar prior-β grains extend to multiple layers, making their grains longer, but their quantity does not increase significantly. According to the Hall–Petch relationship, compared with the direction parallel to the build direction, there are more columnar prior-β grain boundaries perpendicular to the build direction, which hinders the dislocation slip and increases the strength of the sample.

**Figure 12:**
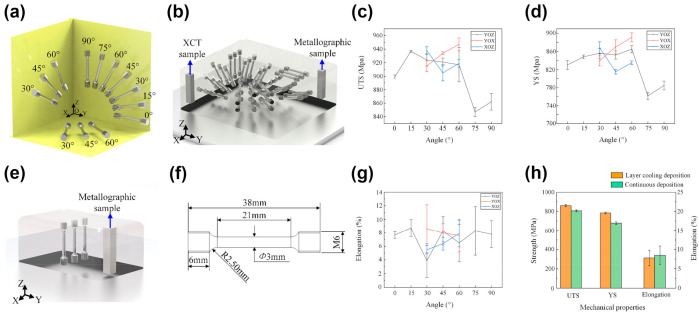
Comparison of different orientations for the preparation of additively manufactured metal materials and their mechanical properties. (**a**) Tensile specimens prepared at varying angles from disparate surfaces are employed for the purpose of testing the anisotropy of mechanical properties; (**b**) schematic diagram and metallography of the mechanical anisotropic tensile sample in intermittent interlayer cooling, and schematic diagram of the XCT sample; (**e**) schematic diagram of the mechanical anisotropic tensile sample and metallographic sample in continuous deposition; (**f**) sample sizes in the reference; (**c**,**d**,**g**,**h**) mechanical properties (reproduced with permission from Ref. [106]. Copyright (2022), Elsevier).

In addition to the conventional BD (building direction), TD (transverse direction), and ND (normal direction), Xu et al. [106] also studied the mechanical performance anisotropy of Ti22Al24Nb. As shown in Figure 12, the authors investigated the differences in the mechanical properties of the alloy prepared by the interlayer cooling deposition method on different planes (Figure 12a), different angles on the same plane (Figure 12a,b), and compared with samples prepared by the continuous deposition method (Figure 12e). The mechanical test results show that samples deposited by the intermittent layer cooling method exhibit a certain degree of mechanical anisotropy. Meanwhile, samples produced by the intermittent layer deposition method exhibit higher ultimate tensile strength (UTS) and yield strength (YS) but lower elongation rates than those produced by the continuous deposition method. This is due to the difference in deposition methods leading to different grain morphologies. The β-grain morphology of samples prepared by the interlayer cooling deposition method is mainly a mixture of small-sized, short, columnar grains and equiaxed grains, while the β-grain of samples prepared by the continuous deposition method is a coarse columnar grain. The interlayer cooling deposition method is beneficial for grain refinement, thereby improving the strength of the samples.

### 3.3. Additive Manufacturing-Fabricated Stainless Steel

Stainless steel is widely used in our daily lives due to its excellent corrosion resistance, high-temperature oxidation resistance, high strength at high temperatures, high surface finish, and good weldability, making it suitable for a wide range of applications in food and beverage, chemical, pharmaceutical, medical device manufacturing, construction, household appliances, offshore and shipbuilding, automotive manufacturing, energy, and industrial sectors [107,108,109,110]. Long processing cycles, high processing costs, and long delivery times characterize traditional machining of stainless-steel materials. AM technology, on the other hand, can produce very complex structures at lower costs and with less waste, offering significant economic advantages. In addition to the above advantages, stainless steel is the cheapest metal material compared with other AM materials. Its simple preparation process and good powder formability make it the oldest metal material used in AM. Moreover, stainless steel is relatively complex and comes in various colors, such as silver, bronze, and white, making it commonly used in AM for jewelry, functional components, and small sculptures.

The stainless steels commonly used in AM include austenitic stainless steel [111], martensitic precipitation-hardening steel [112], precipitation-hardening stainless steel [113], and tool steel [114]. The majority of steel materials processed by L-PBF is composed of austenitic stainless steel, martensitic steel, and precipitation-hardening stainless steel. In contrast, the EB-PBF process mainly utilizes tool steels such as austenitic stainless steel (316L). Research also shows the use of austenitic stainless steel (316L) and tool steel (H13) in LMD (laser metal deposition).

### 3.4. Anisotropy in Additive Manufacturing-Fabricated Stainless Steel

Considerable research has, to date, investigated the anisotropy of stainless-steel components formed using AM technology. Michal et al. [115] conducted a comparative study on the anisotropy of the mechanical properties of 316L stainless steel prepared using LENS in different directions. The results showed that, for the same 316L component, the tensile strength and yield strength parallel to the build direction were superior to those perpendicular to the build direction. Yang et al. [116] studied the wear resistance anisotropy of 316L stainless-steel components prepared using SLM, revealing that, compared with the anisotropy of wear under high loads, the anisotropy of wear under low loads is more pronounced, which is determined by its microstructure. Wen et al. [117] investigated the tensile strength and elongation at different directions of 316L stainless-steel components prepared using SLM, showing that the thermodynamic effects during processing can lead to the anisotropy of the mechanical properties of the components. The data in Table 1 also show the objective existence of the anisotropy of tensile performance in stainless-steel components that can be prepared using AM technology [72,73].

**Figure 13:**
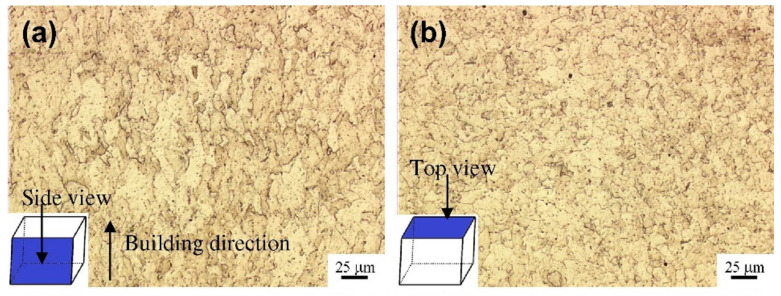
OM observation of the as-fabricated iron cubes (**a**) from the side view and (**b**) from the top view (reproduced with permission from Ref. [119]. Copyright (2014), Elsevier).

**Figure 14:**
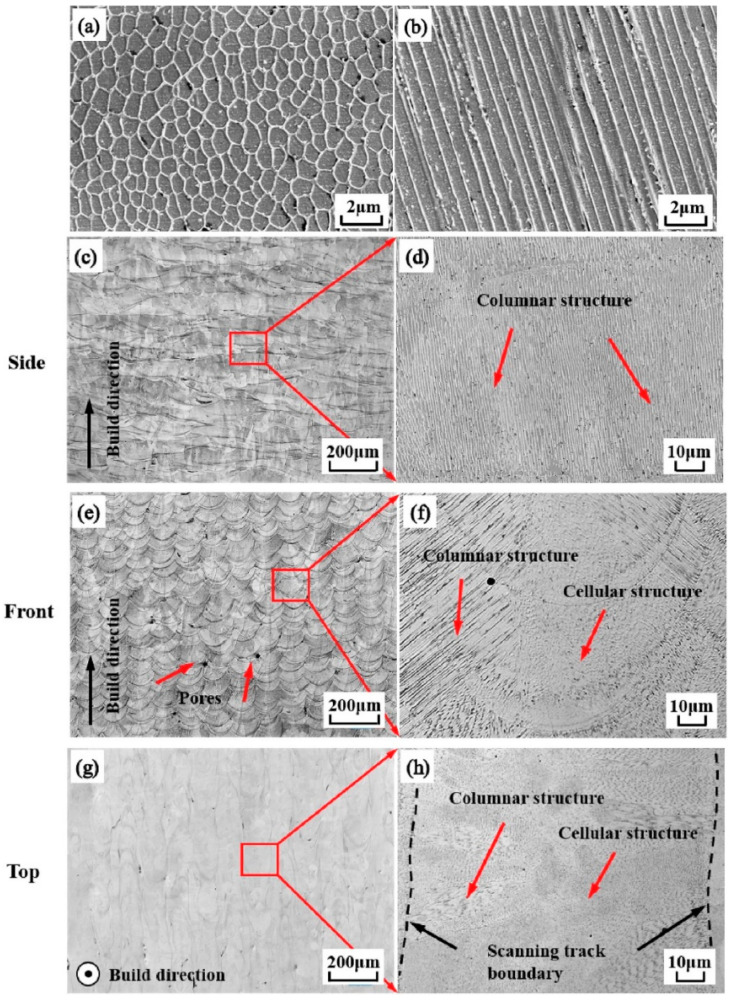
Microstructure images of the SLMed 316L sample: (**a**) cellular structures, (**b**) columnar structures, and (**c**–**h**) microstructures of the side, front, and top surfaces of the SLMed 316L sample and their enlargements (reproduced with permission from Ref. [116]. Copyright (2019), Elsevier).

As shown in Figure 13 and Figure 14, similar to the forming process of titanium alloy components, in the process of preparing stainless-steel components by AM, columnar grains grow through layers from the bottom due to the nearly identical complex thermophysical processes and chemical compositions of each deposition layer [118,119]. On the side surface of the stainless-steel-forming piece (Figure 14b), a layered structure characterized by approximately parallel fusion lines can be observed, caused by the layer-by-layer melting process. From Figure 13 and Figure 14, it is evident that the side surface primarily exhibits a columnar structure aligned parallel to the building direction. This phenomenon occurs as grains grow along the direction of heat flow. In the PBF process, the heat generated by the laser flows from the top to the base, resulting in the vertical growth of crystals. The front surface displays arc-shaped melt pools that are highly arranged along the building direction (Figure 14e). Additionally, due to the residual oxygen in the SLM process, some pores can be observed in the overlapping area of adjacent melt pools. As shown in Figure 13a and Figure 14e, the front surface exhibits columnar and cellular structures. The highest temperature is at the center of the melt pool, so the growth direction of the columnar structure is perpendicular to the boundary of the melt pool. Figure 14g shows the melting tracks on the top surface. These tracks are parallel to each other and are related to the scanning pattern of each layer. The top surface is composed of a large number of cellular structures and a small number of columnar structures. The remelting of adjacent scanning tracks forms the columnar structure around the boundary of the scanning track. Columnar grains are the fundamental microstructure that cause the anisotropy of mechanical properties in stainless-steel components. Due to the presence of columnar grains, for the anisotropy of friction and wear of stainless steel under low loads, the sliding resistance of columnar structures differs in different sliding directions. Hence, their friction and wear performance are different in different directions, while under high loads, the impact of this structural difference is relatively small, thus showing isotropy. For the anisotropy of ductility and strength of stainless steel, the elongated columnar grain structure, the spatial topology of melt pool boundaries [118], and numerous defects in each layer (such as pores at interlayer boundaries) are all reasons for the anisotropy of its ductility and strength.

### 3.5. Additive Manufacturing-Fabricated Mg and Al Alloys

Magnesium–aluminum alloys are widely used in the manufacturing industry due to their excellent lightweight and high-strength properties, meeting the demand for lightweight. They have also become preferred alternative materials for major manufacturers of AM technology. However, compared with the titanium alloys and stainless steel mentioned previously, aluminum alloys have less commercial advantages. For the laser wavelength commonly used in LBM and LMD processes, aluminum has a higher spectral reflectance, making EBM technology more suitable for manufacturing aluminum components using AM technology. In addition to the issues mentioned above, when we used the electron beam to process the aluminum alloys in a vacuum chamber, many solute phases in aluminum alloys have vapor pressure that differ significantly from that of aluminum, causing these solutes to vaporize before aluminum and leading to complex chemical reactions [29,109,120,121].

Despite the challenges in the AM of aluminum alloys, powder and wire forms of aluminum alloy AM materials are still available. This availability is due to AM processes essentially being a form of incremental casting. The most commonly used aluminum alloys for AM are AlSi10Mg [109,122] and AlSi12 [109,123]. Other commercial aluminum alloys include Al-Mg-Si (6061 [77,109,124]) and Al-Cu (2139 [109,125]) alloys. However, it is important to note that, despite the availability of several aluminum alloys for AM, AlSi10Mg produced using LBM technology remains the mainstream choice.

### 3.6. Anisotropy in Additive Manufacturing-Fabricated Mg and Al Alloys

**Figure 15:**
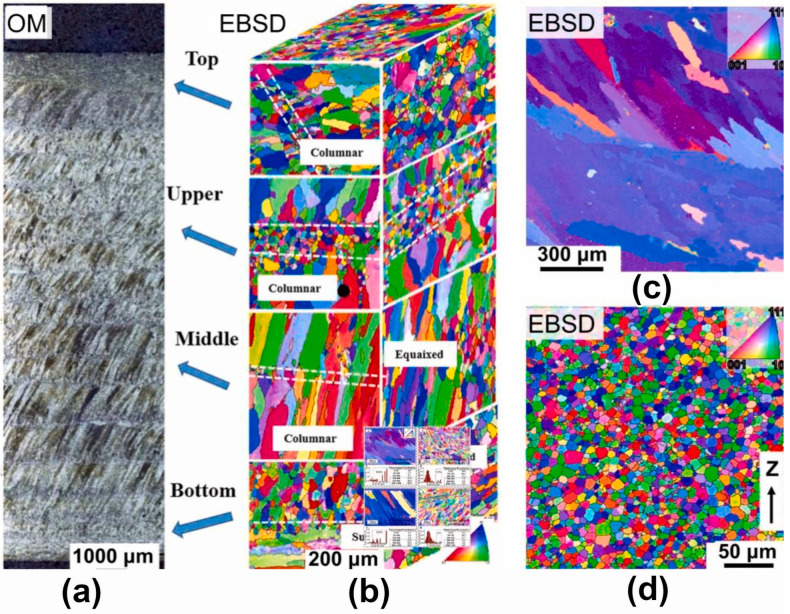
Microstructure of wire-arc AM aluminum alloys. (**a**) Optical microscopy and (**b**) EBSD of AM Al2219 on the water-cooled substrate, (**c**) EBSD of AlZnMgCu, and (**d**) EBSD of AlZnMgCuSc (reproduced with permission from Ref. [127]. Copyright (2023), Elsevier).

To date, researchers have considerably researched the anisotropy of magnesium aluminum alloy components produced using AM technology. Lore et al. [126] indicate that, due to the unique process of L-PBF, magnesium aluminum alloy components manufactured using this process exhibit the growth of elongated grains along the internal temperature gradient of the melt pool, with each layer’s similar chemical composition providing the necessary conditions for layer-by-layer growth. Compared with the growth rate parallel to the temperature gradient, the growth rate of grains in the direction perpendicular to the temperature gradient is almost zero. The research by Idan et al. [74] indicates that the combined effects of directional cooling and repeated thermal cycling significantly impact the microstructure of deposited alloys, leading to a completely different microstructure of AM samples compared with cast samples (Figure 15). The differences in microstructure also directly affect the mechanical properties of AM components. As shown in Table 1, magnesium aluminum alloy components produced using AM technology exhibit some level of anisotropy but with slight differences.

Generally, P-PBF and directional solidification can result in more pronounced orientation differences in the material’s microstructure, with the most favorable growth direction in cubic materials being <100>. Consequently, magnesium–aluminum alloys prepared using L-PBF exhibit a more pronounced texture. Research by Suryawanshi et al. [75] indicates that, in addition to the influence of microstructure, the mesostructure imparted by laser tracks also has a significant impact on the anisotropy of plasticity in aluminum–magnesium alloys prepared by AM. Compared with paths perpendicular to the build direction, crack paths parallel to the build direction exhibit less tortuosity because they experience a relatively flat growth surface. This crack propagation pattern is also one of the reasons for the anisotropic behavior of aluminum–magnesium alloys prepared using AM technology. Similar to titanium alloys and stainless-steel materials, the formation of columnar grains due to differences in temperature gradients in the horizontal and vertical directions during the preparation of aluminum–magnesium alloys is also an essential factor contributing to their anisotropy.

### 3.7. Additive Manufacturing-Fabricated High-Temperature Alloys

High-temperature alloys refer to a class of metal materials that can operate for long periods in high-temperature environments above 600 °C under certain stress conditions. They possess high-temperature strength, good heat corrosion and oxidation resistance, and good plasticity and toughness. Based on the alloy matrix, we roughly divided them into three categories: iron-based, nickel-based, and cobalt-based alloys [41,109]. Compared with iron-based and cobalt-based high-temperature alloys, nickel-based high-temperature alloys are the most widely used in the entire high-temperature alloy field. Chromium–nickel–iron alloy 625 [99] and chromium–nickel–iron alloy 718 [100] high-temperature alloys have been developed and applied in high-temperature applications. Initially, researchers developed high-temperature alloys for the turbine components in turbojet engines, mainly used in high-performance engines. With the development of high-temperature alloys in modern advanced aerospace engines, using high-temperature alloy materials accounts for 40% to 60% of the total engine mass [128,129,130]. The application of high-temperature alloys is not only limited to the aerospace field; they also exhibit excellent performance in high-temperature applications in the power generation industry.

With the progress of the times and the increasing demands on the temperature and performance of high-temperature alloys in the development of modern high-performance aerospace engines, as well as the increasing complexity and integration of nickel-based high-temperature alloy components, traditional processing methods are no longer able to meet industrial applications. The traditional ingot metallurgy process has a slow cooling speed, serious element and second-phase segregation in the ingot, poor hot working performance, high processing cost, uneven structure, and unstable performance [41,131]. Therefore, advanced and efficient AM technology has become a new method to solve the technological bottleneck in forming high-temperature alloys. Currently, chromium–nickel–iron alloy 625 and chromium–nickel–iron alloy 718 are the most commonly manufactured high-temperature alloys using AM processes, primarily used in the industrial field. In addition to these two high-temperature alloys, CoCr alloys are also used in the biomedical field [132,133].

### 3.8. Anisotropy in AM-Fabricated High-Temperature Alloys

The nickel-based high-temperature alloys widely studied in AM include IN625, IN718, Hastelloy X, CM247LC, and IN738LC, among others [76,130,134,135,136,137,138]. IN718, the most widely used precipitation-strengthened nickel-based high-temperature alloy, accounts for over 35% of all high-temperature alloy production and is extensively used to manufacture gas turbine components, turbine blades, and aerospace engine combustors. As a precipitation-strengthened high-temperature alloy, the total content of Al and Ti in IN718 alloy remains relatively low, exhibiting excellent weldability and additive manufacturability. Additionally, the IN718 alloy contains a small amount of γ″ phase and the phase-forming element Nb. Common precipitate phases include γ′ and γ″, δ phase, MX carbide phase, and Laves phase [78,134,139,140].

**Figure 16:**
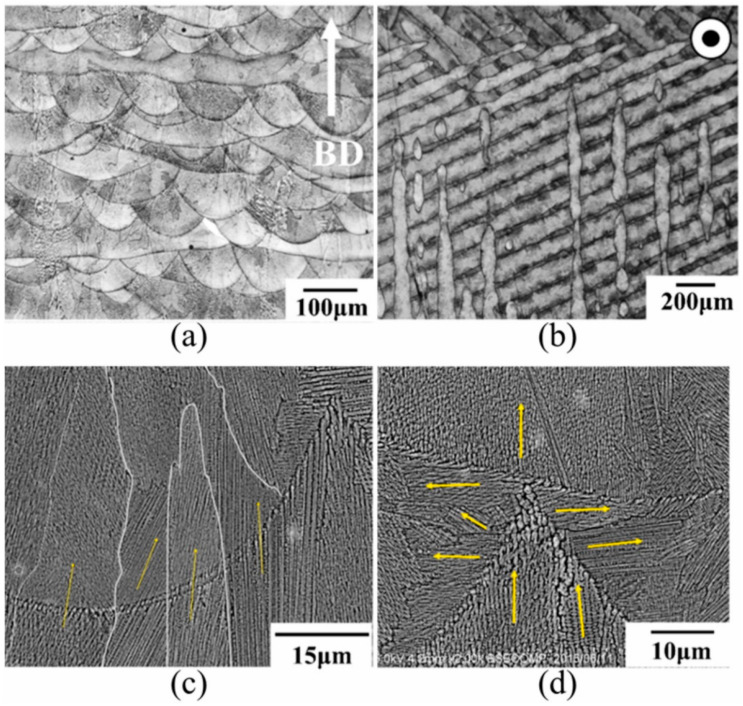
Images of as-built L-PBF Inconel 718 specimens. (**a**) Side view. (**b**) Top view. (**c**) The melt pool boundaries between layers. (**d**) The melt pool boundaries between adjacent tracks from the side. (Reproduced with permission from Ref. [138]. Copyright (2021), Elsevier).

Anisotropy is a common phenomenon in the components of high-temperature alloys prepared using AM technology. Tomus et al. [141] pointed out that the presence of melt pool boundaries is the reason for the anisotropy in Hastelloy X. However, other researchers have also indicated that the anisotropy in strength and ductility of Hastelloy X without obvious melt pool boundaries are related to the columnar grains produced during the manufacturing process and the voids generated due to the lack of melting [33,142]. Figure 16 illustrates the distinctive anisotropic microstructure generated by the PBF process, exhibiting the visible melt pools and laser scanning tracks. In Figure 16a, the arrows indicate the building direction (BD), while in Figure 16b, the circles represent the plane perpendicular to the BD. The melt pools visible in Figure 16a and the laser scanning tracks visible in Figure 16b are indicated by arrows. The yellow arrows in Figure 16c,d represent the growth direction of dendrites. In Figure 16c, dendritic grains grow along the building direction, while in Figure 16d, dendritic grains do not have a preferred growth direction [143]. These findings illustrate the anisotropic microstructure produced by the PBF process.

**Figure 17:**
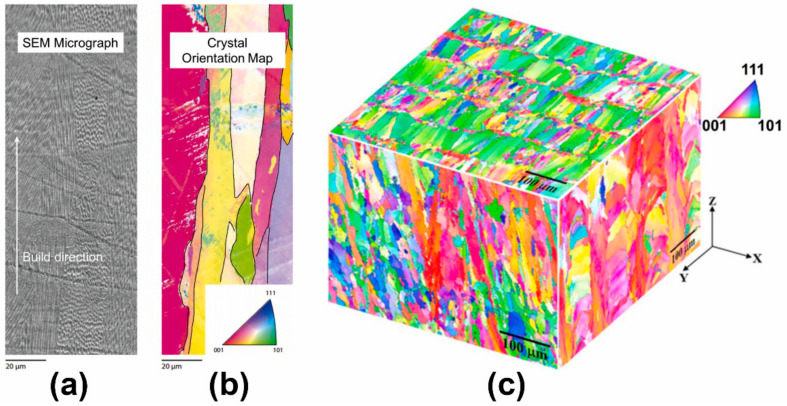
(**a**) Scanning electron microscopy (SEM) micrograph of the cross-section of a Co-29Cr-6Mo alloy produced using the selective laser melting (SLM) process. (**b**) The corresponding crystal orientation map of the micrograph demonstrates the epitaxial columnar grain morphology observed in metal additive manufacturing (AM) parts (reproduced with permission from Ref. [35]. Copyright (2018), Elsevier). (**c**) Electron backscatter diffraction maps of an as-built L-PBF Inconel 718 specimen (reproduced with permission from Ref. [138]. Copyright (2021), Elsevier).

**Figure 18:**
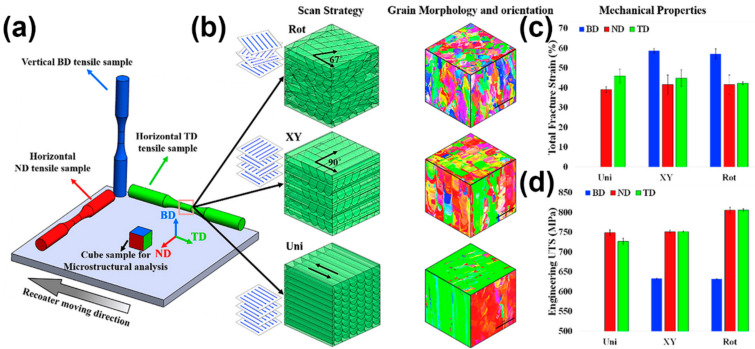
Hastelloy X in three directions of BD(ZXY,ZYX), ND(XYZ,XZY), and TD(YXZ,YZX) obtained from as-build samples with three different scan strategies of Stripe Uni, Stripe XY, and Stripe Rot. (**a**) Schematic representation of tensile and cube samples’ orientation on the build plate. Nomenclature of reference directions for mechanical and microstructural analyses is shown as well. (**b**) Three-dimensional visualization of the microstructure of L-PBF samples with Stripe Uni, Stripe XY, and Stripe Rot scan strategies. The reference direction for each graph is normal to the cross-section. (**c**) Bar plot of ultimate tensile strength (UTS). (**d**) Bar plot of fracture strain. (Reproduced with permission from Ref. [145]. Copyright (2019), Elsevier).

As shown in Figure 17, dendritic grains grow along the build direction, and the sample exhibits a strong <100> texture in the build direction. Small equiaxed grains with an average size of around ten μm are observable in the overlapping region between laser tracks. The microstructural differences in these two directions result in the anisotropy of the nickel-based superalloy composition, leading to anisotropy in its mechanical properties. Different laser scanning strategies can lead to different solidification modes, thus producing different grain morphologies and crystal textures. Keshavarzkermani et al. [144] studied the effects of different scanning strategies on the microstructure and mechanical properties of the Hastelloy X alloy prepared using the L-PBF method. Figure 18a shows tensile specimens made using different scanning strategies, with the build direction (BD), transverse direction (TD), and normal direction (ND) identified. Here, the BD is parallel to the build direction of the tensile specimen, TD is parallel to the machine travel direction, and ND is perpendicular to the machine travel direction. Figure 18b shows schematic diagrams of different scanning strategies and a three-dimensional visualization of microstructures of L-PBF samples, from top to bottom: Stripe Rot scanning with a 67° interlayer rotation, Stripe XY scanning with a 90° rotation, and Stripe Uni scanning without any rotation and bidirectional scanning. The three scanning strategies demonstrate crystallographic orientation differences when viewed from different directions. The EBSD image shows that samples prepared using the Stripe Rot scanning strategy have finer grains and exhibit a fine structure in the ND, TD, and BD directions with random crystal orientations in the microstructure. The intermittent competition of grain growth, caused by the rotating laser irradiation paths in the Stripe Rot samples, accounts for this microstructural refinement. The Stripe XY sample exhibits columnar grains growing along the BD direction and shows a weak cubic texture. In samples prepared using the Stripe Rot scanning strategy, the laser tracks switch between the TD and ND directions; hence, a clear boundary of grain morphology caused by the laser scanning path can be observed in these samples. The finer grain morphology observed on the BD section is due to the growth of columnar grains. Figure 18c,d illustrate the mechanical properties of samples prepared using the three distinct scanning strategies. For L-PBF samples prepared using the same scanning strategy, there is anisotropy in their mechanical properties. The differences in elongation at break in the ND/TD direction are small for samples prepared using the three scanning strategies. For samples prepared using the Stripe XY and Stripe Rot scanning strategies, the elongation at break when prepared along the BD direction is about 14% higher than when prepared along the ND and TD directions. Ultimate tensile strength (UTS) of samples prepared using the Stripe XY and Stripe Rot scanning strategies in the TD, ND, and BD directions is the lowest. Comparing the UTS in the ND/TD direction of the three samples reveals that the Stripe Rot sample has the highest UTS. The different microstructures account for the variations in mechanical properties between different samples and between different directions within the same sample. Due to the presence of large elongated grains in the ND/TD section and fewer grain boundaries normal to the loading direction compared with the BD loading direction, the ductility of the columnar grain structure exhibits anisotropic behavior [141]. Furthermore, the average length of columnar grains along the BD direction is approximately 3–4 times the average width of grains along the TD/ND directions, which results in samples along the BD direction exhibiting higher elongation at break. Compared with samples prepared along the BD direction, samples along the ND/TD directions have finer grain structures, resulting in higher ultimate tensile strength (UTS) along the ND/TD directions. The observed grain morphologies also account for the relative difference in UTS between the Stripe Rot and Stripe XY samples. Different scanning strategies affect the direction of heat flow during the preparation process, leading to different microstructures. Therefore, the scanning strategies and parameters used during the AM process are also important factors contributing to the anisotropy of samples.

### 3.9. Improving the Anisotropy of Additively Manufactured Metallic Materials

The analysis of the anisotropic microstructures of metal components fabricated by the four categories of AM technologies reveals that, due to the inherent characteristics of AM technology, the anisotropy of microstructure and properties in the fabrication of components is inevitable [2,35,57,78,117,118,145]. To improve the performance and service life of components manufactured by AM technology, many researchers have begun to study how to improve the microstructure and properties of components through post-heat treatment and surface processing [119]. Bey et al. [60] found that a certain degree of heat treatment leads to the formation of fine grain structures in TC4 fabricated by the L-PBF process, improving its performance. Zhang et al. [62] indicated that continuous dynamic recrystallization during hot deformation, following phase transformation, primarily forms the new ultrafine grains. Qiu et al. [58,61] demonstrates that most of the voids in the components after forming can be closed by hot isostatic pressing, and the tensile properties of the samples are equivalent to those of samples processed by thermomechanical treatment and annealing. Parts after post-processing, such as surface treatment and heat treatment, are even better than those of cast and forged parts. In addition to post-heat treatment and surface processing, many researchers have also improved the process by optimizing process parameters and workflows to reduce the anisotropy of microstructure and properties in components. Qiu et al. [58] observed that an increase in laser power and scanning speed generally results in a reduction in porosity in components fabricated using the SLM process. Additionally, samples constructed horizontally have higher porosity than samples constructed vertically. Mumtaz et al. [146] also found that using optimized parameters in the SLM process leads to fewer crack formations and higher density in the fabricated samples. As mentioned earlier, many factors affect the anisotropy of microstructure in metal parts fabricated by AM, which are relatively complex. The occurrence of anisotropy can be attributed to a number of factors, including the non-uniform distribution of defects in different directions, the crystal texture, the grain morphology, the microstructural heterogeneity, and other factors [147]. From the process perspective, printing parameters significantly affect the material’s anisotropy, such as laser power, scanning rate, layer thickness, scanning path, powder size, powder shape, roundness distribution, and cooling rate [148]. Therefore, improving the anisotropy of the microstructure and macroscopic properties of metal components fabricated using AM technology and establishing the relationship between equipment, raw material parameters, and microstructure are relatively difficult, and require more experimental and theoretical explorations.

## 4. Summary and Outlook

Additive manufacturing (AM) is a technique for building parts layer by layer and has recently become an option for mass production. To date, several metallic materials, including titanium alloys, stainless steel, magnesium–aluminum alloys, and high-temperature alloys, have been successfully additively manufactured into fully dense parts with excellent properties. Powder bed fusion (PBF) and directed energy deposition (DED) are two of the most commonly employed techniques for the production of metal-formed parts.

This paper presents a summary of the data on the mechanical properties and microstructure of various metals produced using additive manufacturing techniques. Based on this analysis, it can be observed that microstructure and mechanical property anisotropy is a prevalent phenomenon in additive manufactured metal materials. A considerable body of research has demonstrated that horizontally oriented additive manufactured parts exhibit higher mechanical strength than vertically oriented counterparts. The anisotropy in the mechanical properties of formed metal parts produced by additive manufacturing is attributed to microstructural features or defects. Such microstructural features and defects are a consequence of the intrinsic characteristics of additive manufacturing technology. Due to the layer-by-layer nature of additive manufacturing technology, the production of metal parts undergoes a complex thermophysical process, resulting in the metal parts being affected by various processing parameter variables. The melt pool represents the smallest basic unit in the laser additive manufacturing process. Its stability is a guarantee for the stability of the entire process and even the organizational properties of the final formed part. The molten pool typically comprises three distinct zones: the deposition zone, the remelting zone, and the heat-affected zone. The heat-affected zone of the molten metal at the bottom of the solidified metal cyclic input exerts a profound influence on the microstructure of the material, including the grain size and organizational uniformity. Ultimately, this creates to the microstructure of the material and the macro-mechanical properties of anisotropy. The anisotropy of molded parts can be significantly improved by appropriate post-treatment, such as heat treatment.
